# Rethinking the Threats to Scientific Balance in Contexts of Litigation and Regulation

**DOI:** 10.1289/ehp.9988

**Published:** 2007-11-07

**Authors:** William R. Freudenburg

**Affiliations:** Environmental Studies Program, University of California, Santa Barbara, California, USA

**Keywords:** awareness of pressure, ethnography, power relations, scientific biases

## Abstract

**Background:**

Although existing literature does discuss difficulties of doing science in contexts of litigation and regulation, work to date reflects little first-hand experience in such contexts. This gap is understandable but also potentially troubling: Concerns that seem important from afar may or may not match those that are most salient for scientists actually engaged in such work.

**Objectives:**

Drawing on experience on scientific committees and in lawsuits, and using skills developed through doing qualitative fieldwork, I reanalyze past experiences and field notes from the perspective of the 2006 Coronado Conference “Truth and Advocacy in Contexts of Litigation and Regulation.” Although I initially shared the kinds of concerns generally stressed by other scientists and science-studies scholars—emphasizing overt, relatively sinister efforts to limit scientific objectivity—neither the literature nor my initial instincts provided adequate preparation for more subtle influences, which actually created stronger pressures toward bias. Particularly unexpected pressures came from consistent deference and praise for independence and credibility.

**Discussion and Conclusions:**

The cases discussed in this article are by nature suggestive, not definitive; additional research is clearly needed. Future research, however, needs to focus not just on pressures toward bias that are easy to imagine, but also on those that are easy to overlook.

Most scientists are well aware of the need to guard against potential sources of pressure toward bias, particularly when work is conducted in contexts of litigation and regulation, but it is not clear that the usual sources of concern are actually those that are most important. In this article, I argue that, although overt pressures to slant findings may well be problematic, more attention needs to be devoted to the insidious but potentially more significant pressures toward bias that go largely unnoticed, often because they come from unseen or unexpected directions.

I present the argument in four main sections: In the first I discuss my experience on a scientific review panel, illustrating that potential sources of bias in science are more complex than is often assumed. In the second section I discuss that experience and this article’s larger points in the context of existing professional literature on the topic, noting that the literature offers valuable contributions but also includes important oversights and omissions. The third main section illustrates this point by drawing on another, more recent experience, in which I was able to observe first-hand one of the ways in which a major multinational corporation was actively seeding the scientific literature. Finally, the fourth and closing section offers an initial or draft typology of key ways in which the unseen sources of potential bias may be considerably more significant than those that are seen and/or actively resisted.

## Biases—Seen and Unseen

The committees of the National Academy of Sciences/National Research Council (NAS/NRC) generally begin with closed-door sessions, in which committee members are asked to disclose and discuss their sources of bias or conflicts of interest, including any business, research, or other interests or positions that might be perceived by outside observers as creating a potential for a conflict of interest. As NAS staffers commonly explain, the most knowledgeable scientists available on many issues also happen to be the ones who have worked and published extensively on the topics in question, so the intention is not to exclude all scientists who might have strong viewpoints. Instead, the goal is to have a balance of viewpoints and experiences and to discuss openly any such potential sources of bias, real or perceived, at the outset.

Safeguards such as these are laudable, but as indicated by the literature discussed in the next section of this article, they are also incomplete. One of the reasons is illustrated by a bias discussion that occurred in connection with a relatively recent NAS/NRC review of the scientific basis for a proposed low-level radioactive waste facility in the northeastern United States. One after another, the new committee members discussed previous work they had performed for the nuclear industry—and then announced a complete absence of bias, generally doing so with considerable feeling.

Two points about these self-assessments were particularly notable. First, in comparison with the agonizing and painstaking way in which social scientists usually discuss potential sources of bias, the self-assessments were characterized by a remarkable absence of reflection, sensitivity, or in some cases, evidently even awareness of the appearance of potential conflicts, even though the assessments were being offered by scientists who were otherwise highly perceptive. One scientist announced with total conviction, for example, that no one could possibly accuse him of having any biases about the site we were about to review, because he was the top manager for a different low-level nuclear waste site—one that was about a hundred miles away, in the next state. Another announced that, although he had spent some 30 years working in or as a consultant for the nuclear industry—always for organizations that produced nuclear wastes and/or needed to dispose of them—at least 5 years had passed since he had done any work for the specific nuclear contracting companies that happened to be involved with the specific site we were being asked to review. Still another reported that although she had worked extensively for the U.S. Department of Energy—the federal agency with lead responsibility for disposing of high-level nuclear waste—that background had absolutely no relevance to a review of a document that was intended to facilitate the development of a site for low-level nuclear wastes.

So it went, around the table. By the time the discussion had ended, 13 members of the committee—including me—disclosed that we had done work for the companies that generated or attempted to dispose of nuclear waste. Only three members—again including me—had done work for the communities, states, or nongovernmental organizations that were opposed to nuclear facilities in their areas.

In the most diplomatic tones possible, I raised the issue of the appearance of balance. I assured my colleagues that, in the process of working with them, I was already learning to have great respect for their intelligence and integrity. Still, I reminded them, the key issue in the end would have to do with our committee’s credibility—particularly in the eyes of people who, unlike those of us in the room that day, would not have the opportunity to become acquainted with the committee members in person.

This was no minor issue. The site faced intense opposition—much of it animated by claims that previous work on the repository had been performed by agencies and consultants having far more commitment to the nuclear industry than to fair or balanced science. If that original work were to be given a clean bill of health by a committee consisting largely of persons with long histories of employment in that same nuclear industry, the net result might do more to damage the credibility of the committee, and possibly the NRC itself, than to improve the credibility of the work being reviewed.

Several other members of the committee shared this concern, and several did not, but the conversation only became heated at the next step, when we tried to identify potential committee members who might have as much credibility in the eyes of industry opponents as the initial committee members could be expected to have in the eyes of nuclear industry supporters. Although several of the potential candidates were exceptionally well-qualified scientists, virtually all were denounced, most with a level of passion that bordered on ferocity. A typical verdict—offered with a memorable sneer, and with a degree of scorn that goes well beyond what the printed word can convey—was that Professor X was “one of those ‘anti’ types.” Most other potential candidates for the committee were denounced with similar intensity, and the end result was that committee’s composition, although far from being balanced, remained completely unchanged.

In the end, fortunately, the committee managed to finish its work without creating credibility problems for the NAS/NRC. No small part of the reason was that the committee genuinely did carry out its work in a fair and balanced manner, although another part of the reason had to do with the specific conclusions reached. As the committee looked into the methods and calculations that had led to the proposed waste site, signs of increasingly serious errors came to light, and ultimately, the committee unanimously endorsed a report that was highly critical. Although we found no evidence of evil or malicious intent, the final report clearly indicated that the committee saw the analysis and conclusions as fatally flawed.

Both halves of that committee’s experiences are directly relevant for understanding the pressures on science in contexts of litigation and regulation. On the one hand, the scientists on that committee—like most others—truly did think of themselves, with good reason, as being scrupulously fair and balanced, and they would have rebuffed, vigorously, any overt efforts to buy or bias their conclusions. At the same time, however, that committee’s members—almost certainly including me—also shared with other scientists a significantly lower level of ability to recognize, let alone to resist, potential sources of bias that are harder to detect.

A comparable pattern is evident in the common story about a boxer who loses a match not so much because he let his guard down, but because a punch came from an unexpected direction, and he “didn’t see it coming.” For science in contexts of litigation and regulation, it may well be important to focus not just on the obvious ways in which scientists need to be careful of not letting down our scientific guard, but also on the more subtle, gradual, yet perhaps ultimately more powerful ways in which bias can creep into scientific thinking and conclusions, precisely because even the best-intentioned of scientists can fail to see it coming. After a brief review of the academic literature that has emerged on this point, I examine the issue with greater specificity, illustrating the potential dangers by using another first-hand experience that gave me an opportunity to observe such a process at work.

## Past Literature

For readers who are not already familiar with existing analyses of relationships between economic interests and science, a useful starting point is to recognize that the possibilities are generally considered worrisome, not reassuring. Although relatively few concerns are raised in some subsets of the existing literature, such as the classic or Mertonian tradition in the sociology of science—where most authors would see corporate intrusions into the orderly progress of science as being inappropriate but relatively rare [see e.g., Merton (1973)]—the more common patterns involve expressions of concern (Dietz et al. 1989; Freudenburg 1996; Gieryn 1983; Kinchy and Kleinman 2005; Krimsky 2000; Lawless 1993; Molotch 1970).

Much of the attention has focused on relatively straightforward ethical considerations, such as the problems of potentially tainted sources of funding for scientific laboratories, or the ways in which an interest in product commercialization might influence applied research, in terms of either overstating benefits or steering away from consideration of potential drawbacks and risks (Kleinman 1995; Kloppenburg 1988; Levins and Lewontin 1985). One of the most commonly noted and readily understood problems, for example, involves cases where corporate interests have sought to keep unfavorable evidence from coming to public attention or to undermine the legitimacy of more critical work (Dietz et al. 1989; Krimsky 2000; Martin 1999; Rosner and Markowitz 1985). Other analyses, however, have pointed to problems that are less overt. In an analysis of logging on public lands, notably, Hirt (1994) identified a long-standing pattern that he called a “conspiracy of optimism,” involving estimates about the rate at which new trees would grow up to replace the ones removed by logging. My colleagues and I have referred to a comparable pattern in less colorful language, calling attention instead to what we have called the “asymmetry of scientific challenge” [see e.g., Freudenburg (2001); Freudenburg and Gramling (2002); and Freudenburg and Youn (1999)].

As Hirt (1994) noted, such patterns may be especially likely when the relevant government agencies have come to see their interests as being shared with those of an organized industry, as in the case of the Atomic Energy Commission and the nuclear power industry, or of the U.S. Forest Service and the logging industry (Clarke 1985; Martin 1999). In recent years, however, a similar problem has been identified in biomedical or health-related research. As noted by Brownlee (2004), there have been growing concerns about potential biases in these fields, due in part to the fact that private funding of drug trials has grown so spectacularly—from $26 million in 1984 to $2.3 billion in the year 2000—with 60% of the clinical trials now being funded by biomedical companies rather than by the government.

In a pattern with an uncomfortable similarity to Hirt’s “conspiracy of optimism,” one of the most striking implications of this trend has involved what Brownlee (2004) calls “happy talk” about medical products. In one case, for example, Pfizer, the manufacturer of the prescription painkiller Celebrex, supported a study comparing that drug against the over-the-counter medicines ibuprofen and aspirin. The results of a massive 6-month study that favored Celebrex were published in the prestigious *Journal of the American Medical Association* (Silverstein et al. 2000). By contrast, when the final year-long study led to less cheerful findings—showing the sponsor’s painkiller to be associated with more gastrointestinal side effects and 3 times as high a level of serious heart problems than the over-the-counter medicine ibuprofen—those results were never published at all. By the end of 2004, the National Cancer Institute halted a Celebrex trial because high doses of the drug were linked to a tripling of cardiovascular problems—a pattern that Pfizer called “unexpected,” even though one of Pfizer’s own studies had suggested comparable problems (Henderson 2005). As these words were written, Celebrex was the only one of the so-called Cox-2 inhibitors still on the market in the United States, with others having been removed under a cloud of legal and scientific suspicion that included a rare “expression of concern” in the *New England Journal of Medicine* about evident withholding of data on heart attacks related to another Cox-2 drug, Vioxx (Curfman et al. 2005; Girion 2005).

In his more extensive assessment of biomedical research, involving more than 60,000 articles from more than 175 journals, Krimsky (2003) found that this pattern may not be an isolated one. In fact, only 0.5% of the authors revealed potential conflicts of interest, even though roughly a quarter of biomedical researchers were receiving industry funding at the time. Another assessment—a meta-analysis in the *Journal of the American Medical Association* by Bekelman et al. (2003), pulling together results from eight other articles that collectively assessed a total of 1,140 studies—found additional support for the “happy talk” hypothesis, namely, a clear and statistically significant association between industry sponsorship and pro-industry conclusions. Bekelman et al. (2003) also found that roughly a quarter of investigators had industry affiliations, that roughly two-thirds of those investigators’ academic institutions held equity in startup companies that sponsored research being done at the same institutions, and that industry sponsorship was clearly associated with restrictions on publications and data sharing.

## The Internal Compass and the Golden Rule

These and other assessments show that there is a certain degree of legitimacy in the usual concern, which has to do with what might be called the cynical version of the Golden Rule—those who have the gold make the rules. At this point, however, I return to a first-person account—one that starts by acknowledging that this usual concern was also the first one that occurred to me when I had an unexpected opportunity to gain first-hand insights into the potential pitfalls of litigation-related research [for a more extensive discussion, see Freudenburg (2005)]. On the basis of what I learned from my own experiences, I now see other concerns as more worrisome, and I attempt to illustrate here the reasons why that is so.

In essence, my first worry at the time was that I might be agreeing to a Faustian bargain—selling my scientific soul, with the role of the devil being played by company lawyers who in the end would tell me what to publish. What I experienced, I now believe, was just the opposite—not that the corporation ever put strong or even mild pressure on me to publish something with which I would be uncomfortable, but instead, consistent praise for the fact that they considered me to be such a principled, credible scientist. The problem I failed to see, at the time, involved the temptation to start changing my own judgments, in far more subtle ways, in response to their repeated insistence that it was precisely my independence and scientific credibility that they valued.

It all began when I picked up the telephone. At the other end was a gentleman I had not previously known but who worked for a company I knew reasonably well. Over the next several months, he and the experience would teach me a good deal more about one of the ways in which economic interests might influence the course of science—one that had never before occurred to me or, for that matter, to even the most radical and conspiratorially oriented of my students. Given that I had conducted extensive fieldwork and had the habit of recording my notes on a hand-held tape recorder, I followed the same instincts after this telephone call, and there is no better place to start the reporting of what I learned than with the notes I recorded then:

I just got off the telephone with [identifying reference]. He was calling me in conjunction with [his company’s] appeal of the punitive damage awards in [a lawsuit]. . . . [H]e said, he wanted to see if I might be interested in writing an article that [his company] would be able to use as part of its appeal of the punitive damages in the case. As he put it (the following is as close as I can get to a verbatim recording of his remarks from just a few minutes ago):

Naturally, we have a range of expert witnesses and so forth, but we find that it’s also helpful to have people working on articles that come out in academic publications. We’ve often worked with economists, for example. A lot of them feel that punitive damage awards are very inefficient, compared to other approaches such as regulation, and naturally, that’s a perspective we’re quite comfortable in supporting. But we’re exploring whether we might want to work with professors in publishing things from a few other perspectives, too. . . .

Basically, what we’re exploring is whether it’s feasible to get something published in a respectable academic journal, talking about what punitive damage awards do to society, or how they’re not really a very good approach. Then, in our appeal, we can cite the article, and note that professor so-and-so has said in this academic journal, preferably a quite prestigious one, that punitive awards don’t make much sense.

At this point, he and I spent some time exploring various possibilities that might be of interest both to me and to his company. By the end of this conversation, I was greatly intrigued, but also ambivalent. I had qualms about doing this form of consulting work, but I found that my qualms were calmed enough—both through ongoing interactions with this caller and through learning what it was that I was or was not asked to do—that those qualms never became a real roadblock. In essence, I proposed only those topics that I would feel comfortable in turning into journal submissions, leaving to him and his company the question of what to support and pursue. Similarly, although he suggested several topics to me, he never pressured me to take on a topic that seemed to me to be inappropriate. Powerfully counterbalancing the ambivalence, at the same time, was the fact that I was very curious to learn more. From the notes:

I was exploring something I still don’t fully comprehend, save perhaps at a strictly intellectual level—how it is that a company as big as [his] would actually want to pay a sociologist for doing something that we normally think of as providing a useful example of the word “obscure”—publishing in an academic journal—and what good it could possibly do them. . . .

Part of the answer on “what good it could do them” had to do with the dynamics of the appeals process. At the level of an initial or jury trial, the caller explained, academic articles would have relatively little value for his company—a judgment he based in part on research by another one of his company’s consultants. As he summarized that research, ordinary jurors tend to be swayed by nonfactual considerations, including the fact that it is easier to sympathize with “little guy” victims than with a massive corporation such as his, but also, he insisted, “by a kind of lottery mentality . . . they (the jurors) think that ‘next time, that could be me’”—the lucky person who might enjoy a windfall of a similarly huge jury verdict. “Once it gets to the judges,” on the other hand, he said, “you start to have a better shot. . . . With the judges, there’s at least a reasonably good chance that they’ll be able to see things as they ought to be.”

As the process was unfolding, I asked myself the kinds of ethical questions that will occur to many readers of this article. In the end, I wound up concluding in each case that I was in fact proceeding in a scientifically appropriate, ethical way. In retrospect, however, I believe that I was so focused on avoiding overt pressures to state predetermined conclusions that I missed what is now the main point of the present article—I failed to recognize the power of more subtle forms of influence. To be more specific, I was initially worried mainly about an issue that has been raised, eloquently, by one of the reviewers of this article, who argued in his/her review that scientists should establish “a strong rule of thumb: Don’t create academic literature under contract.” At least from that reviewer’s perspective, there is a clear difference between research sponsorship versus preparation of journal articles under contract, because “sponsorship should not allow them [the corporate sponsors] to censor what the researcher writes.” At the outset, my main worry had to do with this very possibility—the potential that the corporate sponsors might try to censor what I wished to write. As events unfolded, I discovered that those sponsors went out of their way to avoid doing anything I might have interpreted as raising even vague hints of censorship—and yet in the end, that did not prevent the relationship from having a far greater influence over my thinking and writing than I believe I was able to recognize at the time.

My thinking at the time is perhaps best-illustrated by an excerpt from field notes that, with the benefits of hindsight, I now see as being insufficiently self-critical or thoughtful, even though I remember seeing myself as having been concerned with balance when I wrote these words:

I must admit, I have quite a complex set of reactions at the moment. Part of me is deeply bothered by the fact that this sort of thing is going on—at all—let alone by the fact that I might become part of it. Another part of me—the middle-of-the-road part—is tapping me on the shoulder, reminding me that I’ve always said I try to be a straight shooter, I call them as I see them, and whether it’s [his company, or his company’s] sworn enemies, if they can use my stuff, fine, and if not, that’s their choice. . . .

I see a clear potential for ethical quagmires and quicksand, of the bottomless-pit variety, but I guess at least for the moment, so long as I continue to be worried about those questions, there’s at least some reasonable hope that I’ll continue to learn more, while not completely selling my soul. . . . I guess I simply need to remain true to my ethnographic principles, but also my researcher principles . . . performing a remarkable balancing act at the same time—giving [his company] a quality product for the money . . . while not sending anything off to a peer-reviewed journal that I’m not comfortable signing my name to.

Soon after that, his company flew me down to their headquarters for a face-to-face meeting. At that meeting, I remember saying to them that, although I had written some articles that would warm their hearts and others that would be more likely to bring them heartburn, the ones they had in front of them before I arrived were mostly of the “heartburn” variety. Under the circumstances, I wondered, why had they invited me down anyway? Their answer, offered without hesitation: “How do you suppose we could find somebody credible who hasn’t said some critical things about us?” That response, and the good-natured way in which it was presented, did a good deal to put me at ease, as did the congenial tone of our conversations more broadly. They made it clear that day—and reinforced the point in many ways in subsequent interchanges—that they had absolutely no intention of censoring my work. They never did censor my work, or even drop vague hints that they might be so inclined. Instead, they emphasized repeatedly that they saw me as the kind of principled, independent scientist who could never be swayed by nonscientific factors such as threats or dollars. The problem seems to have been that, each time they offered such assurances, I came to believe them more.

By the end of that visit, we agreed that we would examine several different possibilities, and that I would work to develop one or more of those ideas for an article. Over the next several weeks, I did in fact develop several outlines for potential article submissions. The one that the company found most interesting had to do with some thoughts I had already “been thinking about writing up some day,” arguing that the adversarial approaches of the legal system ran precisely counter to the prescriptions for sensible risk management that were beginning to emerge from the literature on risk analysis and risk management at the time. In essence, although adversarial procedures encourage secrecy, the findings from the literature on “highly reliable organizations” were beginning to suggest the importance of “organizational permeability” and other forms of openness, for improving organizational performance in general and risk management in particular (Clarke 1993; LaPorte 1996; LaPorte and Keller 1996; Shrader-Frechette 1993). I did a quick writeup of a draft paper and sent it to my contact at the company.

Neither my contact nor anyone else at his company expressed any strongly negative reactions, although at least one of the company’s lawyers did point out later that more openness could be bothersome for his corporation—it could increase the number of people who would know enough about the company to be able to sue it. The larger problem, as my contact ultimately explained to me—in an explanation that I believe to have been genuine—simply came down to the value of my argument to his company. He thought the article would be “nice,” he said, but it would not really help their case enough to be worth spending the additional dollars that would be required, at my consulting rate, to turn it from a draft into a published article.

I stress again at this point that, far from raising any hints about censorship at that time, he made a point of encouraging me to submit the article to a journal if I wished to do so—his was a concern not about the content of the paper, but about paying me to work on it. At the time, I remember thinking that his reaction was similar to a rating of “good” on a grant proposal to the National Science Foundation: Even if a reviewer might check the box that says, “Fund this proposal if resources are plentiful,” few scientists have ever encountered cases where resources are that plentiful. I need to add that my own evaluation was not that different from his: Even after all these years, I still have not invested the time that would be needed to rewrite that draft paper and submit it to a journal. It remains buried in a file cabinet, and unless I encounter some new reason to revise and refine that early draft paper, it may ultimately be less likely to go into a journal than to go into university recycling bins.

We did discuss other possible article topics before parting company, but my reactions to his suggestions were no more enthusiastic than were his reactions to mine, in large part because his company was interested in articles concluding that punitive damages were irrational and “out of control,” and the actual research findings on that point tended not to support their preferred conclusion [see e.g., Eisenberg (2001) and Galanter (1983)]. After a few more conversations, but in quite a cordial fashion, we agreed to a parting of ways. He closed by reminding me that he fully intended to pay me for the hours I had put in, and I reassured myself that I had indeed remained true to my principles, because I never did allow the potential for income to tempt me into writing something that I felt to be inconsistent with the available evidence.

## Discussion

Most readers of this article are likely to be familiar the old story about the boiling frog—the observation that a frog will leap out immediately if it is dropped into a cauldron of boiling water, but that it might not even notice (and might thus boil to death) being placed into cool water that is warmed up only gradually. That, however, may not be quite the right metaphor. The larger problem, instead, may have to do with needing to ask how we can see what it is that we fail to see. Part of the answer may lie in being able to think more carefully about the reasons for failing to see something. Just as a magician can make things seem to disappear by getting the audience to focus on something else (Freudenburg and Alario 2007), part of the answer may be that it may be easier to influence scientists’ thinking by praising their independence than by seeking to limit it.

Based in part on the first-hand experience summarized in this article, I now rarely question scientists who claim that they have never been subjected to (overt) pressure to change their findings, or that they are genuinely proud of just how independent they (honestly) believe they are. Although I believe they are doing their best to tell the truth about their own perceptions, however, I am less ready to believe that those perceptions will offer completely reliable information about their actual levels of scientific impartiality. The concern in some ways parallels the issue of “gifts of nominal value”—a specific and specialized quotation that nevertheless generated more than 32,000 “hits” in a Google search performed August 2007. By far, the majority of the “top” hits endorsed the appropriateness of small gifts, in contrast to anything involving greater economic value. As noted in a biomedical context by Kupersanin (2002), however, “many studies have shown that even minor gifts have an impact on clinical decisions.” Despite this commonality, however, my concerns are more specific, relating to the reason why I am less sanguine than the reviewer quoted above about the distinction between corporate support for research versus corporate support for the writing of journal articles.

Somewhat ironically, my revised reasoning is associated with a less cynical version of the Golden Rule, involving the norm of reciprocity and, more specifically, the forms of reciprocity that do not necessarily even involve money. If the companies that support our scientific research are very careful to respect what is important to us in nonfinancial terms—namely, our objectivity and independence—then it may seem only fair for us to ask what we can do for them, “without compromising” our objectivity.

In some ways, such accommodations are little different from the ones we all make every day, when a colleague asks for extra help on a manuscript or a student is in extra need of advice. In contexts of litigation and regulation, however, the consequences can be much more substantial—and as in the case of the NAS/NRC bias discussion that I summarized at the start of this article, the resultant drift of scientists’ self-perceptions can have a cumulative impact that is significantly more systematic in one direction rather than another.

Given that self-perceptions are inherently personalistic, I am offering this article’s warnings not as hard-and-fast rules that deserve to be seen as established but in the form of possibilities that deserve closer attention in the future. As a way of adding specificity to my overall concerns, and potentially providing clearer guidance for future research, Table 1 identifies five more specific categories of pressures toward bias that may deserve greater attention. To switch metaphors, Table 1 suggests that if it is useful to think of science in any context as being a hunt for truth, there are at least five ways in which the unseen hazards or pressures toward bias may be more insidious or more dangerous than the overt hazards that most scientists have learned to expect. The first has to do with each scientist’s own internal sense of direction. The second concerns the mental maps that tell each of us where to look. The third involves the rules by which we believe we are hunting, and the fourth concerns the broader “region” or context in which we believe we are hunting. The fifth set of pressures, finally, pertains to the ways in which we ultimately measure or assess whatever quarry we “bag.” Of all these pressures, perhaps the fifth and final set of pressures is the one that is in greatest need of further discussion [see also Freudenburg et al. (in press) and Michaels and Monforton (2005)].

One of the potential blind spots of science relates back to statistical training. The problem is that, at least until very recently, few scientists learned much about statistical power—the likelihood of failing to recognize a pattern that is actually present. Partly for that reason, even well-known and careful scientists will often emphasize that a “finding” may be statistically significant through chance alone while overlooking the equally true point from the other side of the coin—the fact that even a “statistically insignificant” pattern may nevertheless be substantively important.

A major source of the problem has to do with the difference between “pure” and “applied” science, although this difference, again, is largely unrecognized. In a world of pure science, it can be prudent to concentrate mainly on type I errors and “significance.” The salient risk in such work is that if scientists are not sufficiently careful—that is, careful about type I risks—other scientists may waste time disproving a hypothesis that is simply a random fluke. For decisions that involve real-world risks, on the other hand, the most important risk may well be just the opposite one—the risk of assuming that a chemical compound or a technology is “safe” when in fact it is not. At least in real-world regulatory and/or litigation-related debates, however, regulators rarely ask whether we know enough to allow the public to be subjected to unknown risks [the type II or “statistical power” concern—see Freudenburg et al. (in press)]. Instead, as suggested in Table 2, even among those who have reasonably sophisticated scientific training, the more common tendency has been to ask whether the scientific findings are strong enough to justify the imposition of regulations in the absence of definitive “proof.”

A relatively new line of work on this problem, drawing on the literature in the sociology of science and technology, responds with a four-part argument. First, science is capable of offering only three kinds of answers—yes, no, and maybe. Second, contrary to the widespread assumption that science is neat and definitive—in line instead with the common finding in the sociology of science that it is not—most scientific work in contexts of litigation and regulation falls into the “maybe” category. In the relatively few cases where the evidence becomes clear enough that the parties stop fighting over a given question, in other words, the battles usually just move on to the next questions that are still in the “maybe” category. Third, in most real-world cases of conflicts over litigation and/or regulation, the net result is that victory goes to the side that wins when the answer is “maybe.” Fourth and finally, even well-trained scientists are often remarkably unaware of this pattern. The net result is a reasonably consistent (and generally but not always helpful) scientific tendency to do work that will permit clearer yes/no answers—clear support or rejection of whatever hypotheses are currently being debated—rather than focusing on what may be a more important question in contexts of litigation and regulation, namely, how decisions could be made more rationally and even-handedly in the absence of just such definitive answers.

When I began to think back to the colleagues on the NAS/NRC committee who were so adamant about their freedom from bias in assessing a low-level waste site, my first reaction was the one mentioned in the opening pages of this article—a sense that those colleagues were not being sufficiently thoughtful about potential sources of bias. Having spent more time reflecting on the matter, I think today that all scientists, myself included, may have more resemblance to the colleagues on that committee than I originally recognized. All of us seem to believe, with good reason, that we do a good job of resisting overt or flagrant pressures toward bias, but such clear-cut cases may be far more rare than we often assume. Instead, those clear-cut cases may have more than a passing resemblance to simplified Hollywood villains—the ones that wear black hats may be the easiest to recognize, but they are rarely encountered except in fiction.

At the same time, we often learn that many of our problems and challenges are at least partly of our own making—and partly due to problems that we “didn’t see coming.” A recently deceased but much-beloved colleague used to joke that he specialized in “pointing out the hidden assumptions in other people’s arguments,” and of course, the rest of us often do so as well. For the future, on the other hand, we may all do a better job of recognizing and dealing with threats to scientific balance if we begin to devote more attention to the ways in which scientific rigor and balance can be undermined—in our colleagues and in ourselves—precisely at those times when we see no black hats, and when we are being praised for our balance and integrity instead of feeling a need to defend it.

## Figures and Tables

**Table 1:** Scientists’ abilities to detect/resist “expected” versus “unexpected” sources of bias in the hunt for truth.

Sources of bias	Expected/watched for/resisted	Often unseen, unresisted
Internal sense of direction	Threats; overt pressure to slant findings/conclusions	Congeniality, support; compliments on neutrality
“Maps” of where to look	Lawyers expected to be adversarial (whereas science is collegial)	Lawyers good at changing questions— which are more important than answers
Rules of the “hunt”	Focus on “facts,” on answering questions	Broader “frames” that shape initial selection of questions
Broader region in which the hunt takes place	Economic interests may have purchased media outlets, “bought” journalists	“Subsidies” to mass media from public relations firms, semi-independent journalists
Ways of assessing quarry	“Sound science,” statistical significance	Balanced science—statistical power; trade-offs between type I/type II errors

**Table 2:** Two ways to be wrong in science.

	Hypothesis: technology is safe	Hypothesis: technology is risky
Reality: technology is safe	Correct	Type I error (usually avoided with 95% confidence)
Reality: technology is risky	Type II error (rarely avoided with even 50% confidence)	Correct
